# Low-Grade Endotoxemia Is Associated with NOX2-Mediated Oxidative Stress and Endothelial Dysfunction in Takotsubo Syndrome: A Cross-Sectional Study

**DOI:** 10.3390/antiox15091124

**Published:** 2026-09-05

**Authors:** Lorenzo Loffredo, Enrico Maggio, Simona Bartimoccia, Vito Cantisani, Antonio Angeloni, Aurora Paraninfi, Paolo Ciacci, Simona Battaglia, Federica Armeli, Ilaria Maria Palumbo, Mariaelena Malvasi, Giancarlo D’Ambrosio, Pasquale Pignatelli, Roberto Carnevale, Francesco Violi, Francesco Barillà, Gaetano Tanzilli

**Affiliations:** 1 Interdisciplinary Department of Well-Being, Health and Environmental Sustainability (BESSA), Sapienza University of Rome, 02100 Rieti, Italy; vito.cantisani@uniroma1.it (V.C.);; 2 Department of Medical and Cardiovascular Sciences, Sapienza University of Rome, 00161 Rome, Italy; enrico.maggio@uniroma1.it (E.M.); pasquale.pignatelli@uniroma1.it (P.P.);; 3 Department of Cardiology and Intensive Cardiac Care Unit, A. Perrino Hospital, 72100 Brindisi, Italy; 4 Cardiology Unit, Paolo Colombo Hospital, ASL ROMA 6, 00049 Velletri, Italy; 5 Sandro Pertini Hospital, 00157 Rome, Italy; 6 Department of Medical and Surgical Sciences and Biotechnologies, Sapienza University of Rome, 04100 Latina, Italy; federica.armeli@uniroma1.it (F.A.);; 7 Department of General Surgery, Surgical Specialty and Anaesthesiology, Sapienza University of Rome, 00161 Rome, Italy; 8 Department of Sense Organs, Sapienza University of Rome, 00185 Rome, Italy; 9 Department of General Surgery, Surgical Specialties and Organ Transplantation, Sapienza University of Rome, 00161 Rome, Italy; giancarlo.dambrosio@uniroma1.it; 10 Sapienza University of Rome, 00161 Rome, Italy; 11 Health Sciences, UniCamillus-Saint Camillus International University, 00131 Rome, Italy

**Keywords:** NADPH oxidase, lipopolysaccharide, Takotsubo syndrome, gut–vascular axis, intestinal permeability

## Abstract

Takotsubo syndrome (TTS) is an acute and reversible heart failure condition characterized by transitional left ventricular systolic dysfunction without obstructive coronary artery disease. Although sympathetic hyperactivation is considered a key pathogenic mechanism, the contribution of gut-derived endotoxemia and oxidative stress is still unclear. Lipopolysaccharide (LPS), an endotoxin of Gram-negative bacteria, may translocate from the gut into the bloodstream and increase oxidative stress through activation of NADPH oxidase 2 (NOX2), nitric oxide (NO) depletion and endothelial dysfunction. This study aimed to evaluate circulating LPS levels in TTS and investigate their association with NOX2 activation, oxidative stress, and endothelial dysfunction. Twenty consecutive patients with TTS and 20 age- and sex-matched healthy controls were included. Within 48 h of admission, fasting blood samples were collected to assess soluble NOX2-derived peptide (sNOX2-dp), hydrogen peroxide (H_2_O_2_), NO metabolites (NOx), LPS, and zonulin. Endothelial function was assessed by brachial artery flow-mediated dilation (FMD). Compared with controls, TTS patients had significantly higher serum levels of sNOX2-dp, H_2_O_2_, LPS, and zonulin, lower NOx and impaired FMD. sNOX2-dp was positively correlated with LPS (Rs = 0.539, *p* < 0.001) and zonulin (Rs = 0.331, *p* = 0.037) and inversely correlated with FMD (Rs = −0.462, *p* = 0.003). NOx correlated negatively with H_2_O_2_, zonulin and LPS. In multivariable analysis, LPS was the only independent predictor of FMD (β = 0.498, SE = 0.126, *p* = 0.001) and sNOX2-dp (β = −0.572, SE = 0.034 *p* < 0.001); FMD (β = −0.347, SE = 0.455, *p* = 0.005), H_2_O_2_ (β = 0.445, SE = 0.155, *p* < 0.001), and zonulin (β = 0.298, SE = 2.309, *p* = 0.016) emerged as independent predictors of LPS (adjusted R^2^ = 0.585). TTS is associated with low-grade endotoxemia, NOX2-driven oxidative stress, reduced NO bioavailability, and endothelial dysfunction. The independent association between LPS and NOX2 activation supports a potential gut–vascular axis in TTS pathophysiology.

## 1. Introduction

Takotsubo syndrome (TTS) is an acute and reversible heart failure condition characterized by transitional left ventricular systolic dysfunction without obstructive coronary artery disease [1]. Clinically, it mimics an acute myocardial infarction, with sudden chest pain, electrocardiographic changes, and elevated troponins, but coronary angiography usually shows absence of obstructive coronary arteries [2,3]. Diagnosis of TTS is based on International Expert Consensus criteria and requires exclusion of obstructive coronary disease and myocarditis [4,5,6,7,8]. Although once considered generally benign, TTS is associated with significant acute and long-term complications [9,10] with mortality comparable to ACS [11,12,13]. It mainly affects women, who are more likely to have emotional stress as a trigger, but it can also affect men, who are more likely to experience physical stress as a trigger [14,15].

The pathophysiology of TTS remains unclear. Sympathetic hyperactivation and catecholamine increase are considered pivotal mechanisms [14]. Sympathetic hyperactivation can affect the gut through alterations in gastrointestinal motility [16], splanchnic perfusion [17], and intestinal barrier integrity [18,19]. These changes may, in turn, influence gut microbial homeostasis and facilitate systemic translocation of microbial products, providing a potential mechanistic link between stress-related neuroautonomic activation and gut-derived inflammation. Consequently, elevated catecholamine levels resulting from acute or chronic stress can modify gut microbiota composition and promote intestinal dysbiosis, as stress-induced neuroendocrine changes (including increased norepinephrine and cortisol levels) have been shown to change microbiota, reduce beneficial taxa, and favor the growth of pathogenic bacteria in animal and human studies [20,21,22]. Thus, in such conditions, lipopolysaccharide (LPS), an endotoxin of Gram-negative bacteria, may translocate into the bloodstream and, upon binding to Toll-like receptor 4 (TLR4), promote systemic oxidative stress via NADPH oxidase activation, ultimately contributing to endothelial and platelet dysfunction [23,24]. Among NADPH oxidase isoforms, one of the most studied in the field of cardiovascular disease is the NADPH oxidase type 2 (NOX2) [25]. This enzyme is involved in the host defense innate immune system and in oxidative stress [25]. Its role in cardiovascular disease is closely connected to reactive oxygen species (ROS) overproduction, which favors platelet activation and endothelial dysfunction [25]. Several studies in humans have documented endothelial dysfunction in TTS patients as measured by brachial artery flow-mediated dilation (FMD), which is an indirect functional marker of the NO pathway [26,27,28]. Naegele et al. found reduced FMD in TTS patients compared to healthy subjects [29]. Furthermore, the reduction in troponin peak following the acute phase of TTS took place together with normalization of FMD [30].

Collectively, these experimental and translational observations support the hypothesis that increased circulating LPS may act as an upstream trigger of oxidative stress through activation of NOX2, leading to reduced nitric oxide bioavailability and endothelial dysfunction. However, direct clinical evidence linking circulating LPS, NOX2 activation, oxidative stress, and endothelial dysfunction in patients with TTS is currently lacking. Therefore, the present study aimed to evaluate circulating LPS levels in patients with TTS and to investigate their association with NOX2 activation, oxidative stress, and endothelial dysfunction.

## 2. Materials and Methods

The primary objective of the study was to analyze the role of NOX2-mediated oxidative stress in TTS by measuring circulating levels of the soluble NOX2-derived peptide (sNOX2-dp). Secondary objectives were to (i) assess endothelial dysfunction by FMD; (ii) evaluate the relationships between NOX2 activation and endothelial dysfunction; and (iii) investigate low-grade endotoxemia (by serum LPS) and intestinal permeability (by serum zonulin) as potential triggers and correlates of oxidative stress in TTS.

### 2.1. Study Design and Population

This cross-sectional study was conducted at the Cardiology Department, Policlinico Umberto I (Sapienza University of Rome). Twenty consecutive patients with TTS were enrolled according to the International Expert Consensus Document criteria [6,7].

In patients presenting with chest pain and/or dyspnea and ST-segment elevation or an InterTAK score > 70, urgent coronary angiography with left ventriculography or coronary CT angiography was performed to exclude obstructive coronary artery disease. Red flags for myocarditis (viral prodrome, pericardial effusion, marked inflammatory marker increase) were assessed. When these conditions were excluded, TTS was diagnosed.

Twenty healthy controls were recruited and matched for sex, age, comorbidities, cardiovascular risk factors, and previous cardiovascular disease.

Exclusion criteria were age <18 or >85 years, hepatic or renal insufficiency, acute cerebrovascular disease, acute myocardial infarction, current antioxidant therapy, active infection or malignancy, and active smoking.

### 2.2. Flow-Mediated Dilation (FMD)

FMD was assessed as previously reported [31]. Participants rested supine in a quiet, temperature-controlled room. Brachial artery diameter was measured 3–7 cm above the antecubital fossa; a linear ultrasound probe (Samsung HS30, Samsung, Seoul, Republic of Korea) was used for the assessments. A forearm cuff was inflated to 50 mmHg above the subject’s baseline systolic blood pressure for 5 min, and arterial diameter was measured after reactive hyperemia. FMD was expressed as [(mean brachial diameter after hyperemia − mean baseline brachial diameter) × 100/mean baseline diameter], with mean diameter derived from at least three end-diastolic measurements.

### 2.3. Blood Sampling and Preparation

Blood samples were collected early in the morning from a superficial vein (the antecubital vein) after fasting for at least 8 h, with participants in a seated position. Samples were drawn into BD Vacutainer tubes (Becton, Dickinson and Company, Franklin Lakes, NJ, USA) without anticoagulant. Following collection, blood samples were centrifuged at 300× *g* for 10 min at room temperature (RT). The resulting serum was carefully separated, aliquoted into pre-labeled tubes to avoid repeated freeze–thaw cycles and stored at −80 °C until further biochemical analyses were performed. Samples were stored frozen until analysis and were thawed for the first time specifically for the measurements performed in the present study.

### 2.4. Biochemical Assays

#### 2.4.1. sNOX2-dp Detection

Soluble NOX2-derived peptide (sNOX2-dp), a marker of NOX2 activation, was quantified using a validated enzyme-linked immunosorbent assay (ELISA). The assay was based on a monoclonal antibody specifically directed against an extracellular epitope of the NOX2 protein, corresponding to amino acid residues 224–268. Serum concentrations of sNOX2-dp were determined according to the manufacturer’s protocol and expressed as picograms per milliliter (pg/mL). The measurement of sNOX2-dp provides an indirect assessment of NOX2 activation and has been widely used to evaluate oxidative stress status in clinical and experimental settings. The intra-assay and inter-assay coefficients of variation were 8.95% and 9.01%, respectively.

#### 2.4.2. Hydrogen Peroxide (H_2_O_2_) Measurement

Hydrogen peroxide (H_2_O_2_), a stable reactive oxygen species and a widely recognized marker of oxidative stress, was quantified using a commercially available colorimetric detection kit (Arbor Assays, Ann Arbor, MI, USA). The assay is based on the reaction of H_2_O_2_ with specific chromogenic substrates, generating a colorimetric signal directly proportional to the concentration of hydrogen peroxide present in the sample. Absorbance was measured using a microplate reader according to the manufacturer’s instructions, and H_2_O_2_ concentrations were calculated from a standard calibration curve. Results were expressed as micromoles per liter (µM). The intra-assay and inter-assay coefficients of variation were 2.1% and 3.7%, respectively.

Hydrogen peroxide (H_2_O_2_) levels were measured using a commercially available detection kit (Arbor Assays, Ann Arbor, MI, USA), according to the manufacturer’s instructions. Samples were processed under standardized preanalytical conditions and stored at −80 °C until analysis, minimizing repeated freezing–thawing cycles. Given the potential susceptibility of H_2_O_2_ measurements to preanalytical variability and matrix-related interference, samples were consistently handled and analyzed under standardized analysis conditions.

#### 2.4.3. Nitric Oxide (NOx) Detection

Nitric oxide (NO) bioavailability was assessed by measuring its stable oxidation products, nitrite (NO_2_^−^) and nitrate (NO_3_^−^), collectively referred to as NOx. Serum concentrations of NOx were determined using a commercially available colorimetric assay kit (Arbor Assays, MI, USA), following the manufacturer’s instructions. Briefly, nitrate was enzymatically reduced to nitrite, and total nitrite levels were subsequently quantified through a colorimetric reaction, generating a signal proportional to the concentration of nitric oxide metabolites present in the sample. Absorbance was measured using a microplate reader, and NOx concentrations were calculated using a standard calibration curve. Results were expressed as micromoles per liter (µM). The intra-assay and inter-assay coefficients of variation were 2.9% and 1.7%, respectively.

#### 2.4.4. Zonulin Measurement

Serum zonulin concentrations were quantified using a commercially available enzyme-linked immunosorbent assay (ELISA) kit (Elabscience, Houston, TX, USA), according to the manufacturer’s instructions. Zonulin is a physiological modulator of intestinal tight junctions and is widely used as a biomarker of intestinal permeability and gut barrier integrity. The assay is based on the specific binding of zonulin present in serum samples to immobilized antibodies, followed by enzymatic detection and colorimetric quantification. Absorbance was measured using a microplate reader, and zonulin concentrations were determined from a standard calibration curve generated with known concentrations of the analyte. Results were expressed as nanograms per milliliter (ng/mL). The intra-assay and inter-assay coefficients of variation were <10%.

#### 2.4.5. Serum Lipopolysaccharide (LPS) Measurement

Serum lipopolysaccharide (LPS) concentrations were determined using a commercially available enzyme-linked immunosorbent assay (ELISA) kit (Cusabio, Wuhan, China), following the manufacturer’s instructions. The assay is based on the specific recognition of LPS molecules by immobilized antibodies, followed by enzymatic detection and colorimetric quantification. Absorbance was measured using a microplate reader, and LPS concentrations were calculated from a standard curve generated using known concentrations of the analyte. Results were expressed as picograms per milliliter (pg/mL). The intra-assay and inter-assay coefficients of variation were <10%.

### 2.5. Sample Size and Statistical Analysis

Sample size was calculated for a two-sided Student’s *t*-test based on pilot data demonstrating a mean between-group difference of 12 pg/mL in sNOX2-dp levels (34 pg/mL in TTS patients vs. 22 pg/mL in controls). A minimum of 36 participants in total were required to achieve 90% statistical power to detect this difference at a two-sided significance level of 0.05. The sample size calculation was based on preliminary data obtained from a pilot analysis conducted in a subset of patients enrolled in the present study. Thus, the pilot sample was not independent of the final study population.

Statistical analyses were performed using SPSS 27.0 (IBM, Chicago, IL, USA). Normality was assessed with the Shapiro–Wilk test, and this test showed that all the continuous variables of interest were normally distributed, except for FMD. Normally distributed data are presented as mean ± SD, while not normally distributed data are presented as median [IQR]. Categorical variables were compared using the chi-square test, while continuous variables were compared using Student’s *t*-test or Mann–Whitney U test, as appropriate. Effect sizes were calculated using odds ratios, Cohen’s d, or Rank-biserial correlations, as appropriate. Spearman correlations were used for bivariate associations. The variables of interest with *p* < 0.10 in bivariate analyses were entered into multivariable linear regression using an automated stepwise selection procedure. Multicollinearity among the independent variables was assessed by calculating the variance inflation factor (VIF) and tolerance values. Significant multicollinearity was considered present with a VIF value of maximum 5 and a tolerance value of at least 0.20. Influential observations were assessed using Cook’s distance. Influential cases were identified with Cook’s distance values < 1. A two-sided *p* < 0.05 was considered statistically significant.

### 2.6. Institutional Review Board Statement

The study protocol was approved by the Ethics Committee of Sapienza University of Rome, conducted in accordance with the Declaration of Helsinki, and approved by the Institutional Review Board (Sapienza University–Prot. N. Rif. 7748–Prot. 0836/2024; approval date: 26 September 2024). All participants provided written informed consent.

## 3. Results

Clinical characteristics of TTS patients and controls are reported in Table 1. All enrolled TTS patients were female. No significant differences were observed in demographic variables (age, sex), cardiovascular risk factors (hypertension, diabetes, smoking history, family history of CAD, prior stroke, dyslipidemia), or pharmacological therapies (statin, ACEi/ARB, anticoagulants, beta-blockers) between groups (Table 1). None of the TTS patients had a history of myocardial infarction or prior coronary revascularization.

Regarding the in-hospital course and short-term prognosis, none of the TTS patients required transfer to the intensive care unit, vasopressor support, or mechanical circulatory assistance. At 30 days, no patient experienced rehospitalization, cardiovascular death, or all-cause death.

Compared with controls, TTS patients had significantly higher circulating sNOX2-dp, H_2_O_2_, LPS, and zonulin levels. Conversely, nitric oxide bioavailability (Nox) and FMD were significantly lower in TTS patients (Table 1).

Bivariate analysis showed that sNOX2-dp was positively correlated with serum LPS (Rs = 0.539, *p* < 0.001) and zonulin (Rs = 0.331, *p* = 0.037) and inversely correlated with FMD (Rs = −0.462, *p* = 0.003). NOx was inversely correlated with H_2_O_2_ (Rs = −0.551, *p* < 0.001), LPS (Rs = −0.470, *p* = 0.002), and zonulin (Rs = −0.398, *p* = 0.011). LPS was positively correlated with zonulin (Rs = 0.497, *p* = 0.001) and H_2_O_2_ (Rs = 0.540, *p* < 0.001), and inversely correlated with FMD (Rs = −0.579, *p* < 0.001). Finally, FMD was inversely correlated with zonulin (Rs = −0.433, *p* = 0.005).

In multivariable linear regression analysis, LPS emerged as the only independent predictor of sNOX2-dp (β = 0.498, SE = 0.126, *p* = 0.001), accounting for 24% of the variability in sNOX2-dp levels (adjusted R^2^ = 0.23; standardized residuals: −1.953–2.353; zonulin and FMD were removed through stepwise selection) (Table 2); LPS also remains significant after adjustment for smoking history, hypertension, and dyslipidemia, which showed a borderline non-significant imbalance between groups. In a separate multivariable linear regression model, LPS was also identified as the sole independent predictor of FMD (β = −0.572, SE = 0.034, *p* < 0.001), explaining approximately 31% of FMD variability (adjusted R^2^ = 0.31; standardized residuals: −1.976–2.579; zonulin and sNOX2-dp were removed through stepwise selection) (Table 3); it remains significant also after adjustment for smoking history, hypertension, and dyslipidemia. Finally, in the model including LPS as the dependent variable, FMD (β = −0.347, SE = 0.455, *p* = 0.005), H_2_O_2_ (β = 0.445, SE = 0.155, *p* < 0.001), and zonulin (β = 0.298, SE = 2.309, *p* = 0.016) emerged as independent predictors of LPS, collectively explaining 58.5% of its variability (adjusted R^2^ = 0.585; standardized residuals: −1.942–1.701; NOx and sNOX2-dp were removed through stepwise selection) (Table 4); no evidence of significant multicollinearity (all VIF values < 5 and tolerance > 0.20) and no influential cases (all Cook’s distance values <1) were identified. The same variables remain significant after adjustment for smoking history, hypertension, and dyslipidemia.

## 4. Discussion

In this cross-sectional study, TTS patients exhibited elevated circulating LPS and zonulin, marked endothelial dysfunction (reduced FMD), increased oxidative stress (higher sNOX2-dp and H_2_O_2_), and reduced NO bioavailability compared with matched controls.

Despite growing evidence implicating systemic inflammation, oxidative stress, and endothelial dysfunction in TTS patients, the potential contribution of low-grade endotoxemia to these abnormalities has remained largely unexplored. Previous studies primarily focused on catecholamine excess [32], neurohumoral activation [33], and downstream inflammatory [34] and oxidative pathways [35], whereas the potential role of gut microbiota and subsequent translocation of bacterial products as upstream triggers or amplifiers has received considerably less attention. In particular, studies directly assessing circulating LPS levels and characterizing gut microbiota composition in patients with TTS are lacking. However, Liu et al., in an animal model, showed that stress-induced cardiac dysfunction is accompanied by significant modifications in gut microbiota, including suppression of the growth of the probiotic *Lactobacillus* and an increase in potentially pathogenic bacterial species, such as *Colidextribacter*, supporting the potential involvement of a gut–heart axis [36].

In addition, experimental TTS-like models have explored the involvement of LPS-related innate immune signaling pathways, including the TLR4/NF-κB/ROS axis [37].

Indirect evidence supporting the gut–heart axis hypothesis has also emerged from previous studies reporting increased serum levels of lipopolysaccharide-binding protein (LBP) during the acute phase of TTS [38], as well as from recent proteomic analyses [39]. It should be noted, however, that LBP is prevalently a hepatic acute-phase protein that binds circulating LPS and favors its transfer to CD14, which subsequently delivers LPS to the MD2–TLR4 receptor complex [23,40]. Thus, increased serum LBP may reflect host recognition of endotoxin exposure but may also be influenced by the systemic inflammatory acute-phase response and, therefore, does not necessarily indicate a corresponding increase in circulating LPS levels [41,42,43].

In this context, our finding of increased circulating LPS during the acute phase of TTS extends previous observations of elevated LBP by providing direct evidence of low-grade endotoxemia, rather than solely an indirect marker of the host response to endotoxin [42]. The concomitant increase in zonulin further supports an association with impaired intestinal barrier integrity, although the cross-sectional design does not allow us to establish the gut origin of serum LPS or a causal relationship between barrier modification and endotoxemia. Taken together, these findings support the hypothesis that increased gut permeability and LPS translocation may represent an additional mechanism contributing to increased inflammatory status and oxidative stress in TTS.

A central novel observation is the strong relationship between endotoxemia and NOX2 activation, with LPS emerging as the only independent predictor of sNOX2-dp in multivariable analysis. Low-grade endotoxemia and the gut–heart axis have been described in atherosclerotic and thrombo-inflammatory cardiovascular disease and are mechanistically linked to oxidative stress and endothelial activation [23,44,45]. These data support the hypothesis that LPS may act as an upstream trigger of NOX2 activation and downstream endothelial dysfunction in TTS. Low-grade endotoxemia is also associated with oxidative stress in diseases characterized by endothelial dysfunction, such as coronary microvascular angina [46].

**Figure 1:**
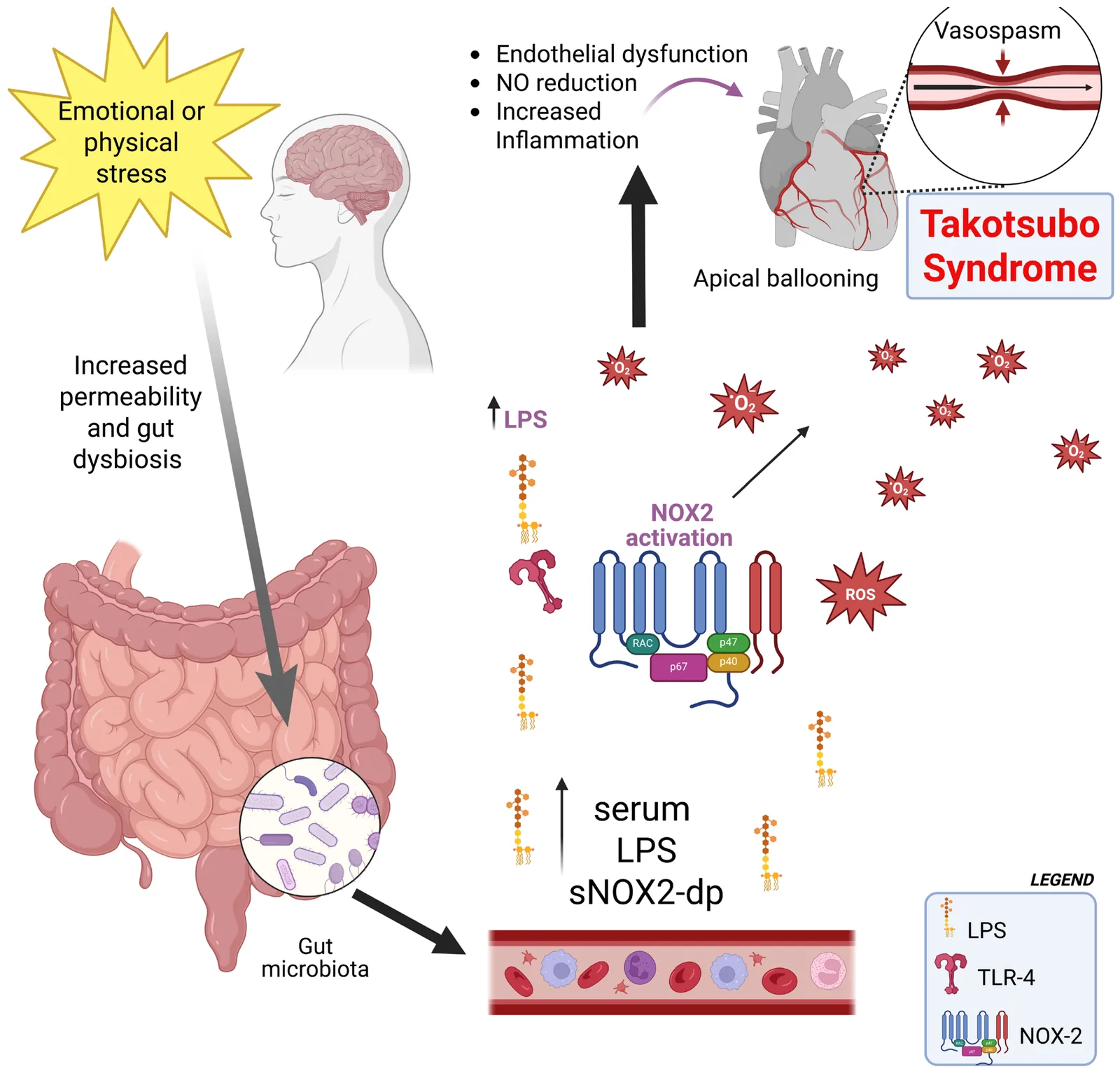
Proposed pathophysiological link between gut-derived endotoxemia, NOX2 activation, oxidative stress, and endothelial dysfunction in Takotsubo syndrome. Emotional or physical stress, frequently observed as a trigger of Takotsubo syndrome, is associated with sympathetic hyperactivation and systemic stress responses that may alter gut physiology. In addition to promoting dysbiosis of the gut microbiota, stress-related changes contribute to increased intestinal permeability (“leaky gut”), allowing lipopolysaccharides (LPSs) from Gram-negative bacteria to more easily translocate into the bloodstream, resulting in low-grade endotoxemia. Circulating LPS activates Toll-like receptor 4 (TLR4) on vascular and blood cells, leading to the activation of NADPH oxidase 2 (NOX2) and subsequent reactive oxygen species (ROS) generation. NOX2-derived oxidative stress reduces nitric oxide (NO) bioavailability, contributing to endothelial dysfunction, impaired flow-mediated dilation (FMD), vascular inflammation, and pro-thrombotic activation. This gut–endotoxemia–NOX2–vascular axis may represent a mechanistic pathway linking stress-related triggers to systemic vascular dysfunction in Takotsubo syndrome.

Endothelial dysfunction found in the present study is aligned with previous analysis demonstrating impaired brachial artery FMD in TTS and a tendency toward normalization during convalescence [29,30,47]. The present findings extend these observations by linking impaired FMD to oxidative stress markers in TTS. Indeed, NOX2 is a major enzymatic source of ROS in cardiovascular settings, and experimental evidence supports NOX isoform-specific contributions to platelet activation and thrombosis [25,48]. Furthermore, FMD showed an inverse correlation with zonulin and LPS. These findings underline the importance of low-grade endotoxemia (at least in part derived from the gut due to increased intestinal permeability) in determining the vascular changes involved in various cardiovascular diseases [44,46,47,49]. In this study, we also observed elevated zonulin levels and a positive relationship between zonulin and LPS, suggesting increased intestinal permeability as a potential contributor to systemic LPS exposure, as observed in previous cardiovascular diseases, such as in patients with microvascular angina [46]. Altogether, these findings could highlight a plausible gut–vascular axis in TTS (Figure 1), which may represent a possible future therapeutic target.

To the best of our current knowledge, this is the first clinical study reporting concomitant NOX2 activation, low-grade endotoxemia, and impaired endothelial function in Takotsubo syndrome. Further mechanistic studies and longitudinal investigations are warranted to establish causality and assess whether modulation of endotoxemia and oxidative stress can improve clinical outcomes. In this regard, in a previous study we demonstrated that treatment with the probiotic *Escherichia coli* strain Nissle 1917 (EcN) significantly reduced circulating levels of lipopolysaccharide (LPS), NOX2 activation, and oxidative stress in treated patients [50]. These effects are likely attributable to EcN’s unique biological properties, including its structurally modified LPS characterized by truncated O-antigen chains [51] and its competitive activity against other Enterobacteriaceae within the gut ecosystem [52,53].

This study has some limitations. First, the sample size was modest and the design was cross-sectional, limiting causal inferences. Second, other NOX isoforms (e.g., NOX1/NOX4) and antioxidant systems (e.g., catalase, superoxide dismutase, glutathione peroxidase) were not measured. Third, the source of circulating LPS was not directly investigated; fecal microbiome profiling was not performed and alternative sources of endotoxemia (e.g., oral inflammation) were not assessed. Fourth, catecholamine levels, central in TTS pathophysiology, were not measured. Fifth, there is an ongoing controversy regarding the specificity of ELISA tests for zonulin. Sixth, sNOX2-dp is a marker of NOX2 activation; however, its measurement does not allow identification of the cellular source of NOX2 activation or direct quantification of NOX2 enzymatic activity. Seventh, the cross-sectional design does not permit causal inference. Finally, we did not investigate the chronic stabilization phase of these patients; therefore, we cannot determine whether the increase in low-grade endotoxemia was confined to the acute phase.

## 5. Conclusions

Takotsubo syndrome is associated with endothelial dysfunction, increased NOX2-mediated oxidative stress, reduced NO bioavailability, and elevated circulating LPS. Low-grade endotoxemia may represent a potential trigger of NOX2 activation and endothelial dysfunction in TTS. These findings warrant further investigation in prospective and interventional studies to confirm these observations and to evaluate the potential contribution of therapeutic strategies targeting oxidative stress and gut-derived endotoxemia in TTS.

## Figures and Tables

**Table 1:** Clinical characteristics and biomarkers of oxidative stress, endotoxemia, and endothelial function in Takotsubo syndrome (TTS) patients and controls.

	TTS (*n* = 20)	Controls (*n* = 20)	*p* Value	Effect Size
Age (years)	67.2 ± 12	62.6 ± 6	0.163	0.45 *
Sex (male/female)	0/20	0/20	–	//
Hypertension (no/yes)	11/9	6/14	0.110	0.351 ^†^
Diabetes (no/yes)	17/3	16/4	0.667	0.706 ^†^
Smoking history (no/yes)	11/9	16/4	0.091	3.273 ^†^
Family history of CAD (no/yes)	18/2	17/3	0.957	//
Prior stroke (no/yes)	19/1	20/0	0.311	0.487 ^†^
Dyslipidemia (no/yes)	9/11	4/16	0.091	0.306 ^†^
Statin use (no/yes)	8/12	6/14	0.507	0.643 ^†^
ACEi/ARB (no/yes)	13/7	9/11	0.204	0.441 ^†^
Anticoagulants (no/yes)	16/4	19/1	0.151	4.750 ^†^
Beta-blockers (no/yes)	5/15	8/12	0.311	2.000 ^†^
sNOX2-dp (pg/mL)	34.5 ± 8.5	23.3 ± 11.3	0.001	1.12 *
H_2_O_2_ (µM)	27.6 ± 7.7	19.7 ± 7.9	0.003	1.01 *
NOx (µM)	24.0 ± 9.0	39.4 ± 17.3	0.001	−1.11 *
LPS (pg/mL)	34.6 ± 10.0	16.9 ± 8.2	<0.001	1.92 *
Zonulin (ng/mL)	2.1 ± 0.6	1.4 ± 0.4	0.002	1.02 *
FMD (%)	1.35 [3.3–0.0]	6.2 [8.7–4.9]	<0.001	−0.805 **

Continuous variables are expressed as mean ± SD or median [IQR], as appropriate, and categorical variables as counts. ^†^ Odds ratio; * Cohen’s d; ** Rank-biserial correlation. Abbreviations: CAD, coronary artery disease; ACEi, angiotensin-converting enzyme inhibitor; ARB, angiotensin receptor blocker; sNOX2-dp, soluble NOX2-derived peptide; H_2_O_2_, hydrogen peroxide; NOx, nitric oxide metabolite; LPS, lipopolysaccharide; FMD, brachial flow-mediated dilation.

**Table 2:** Multivariable linear regression analysis with FMD as the dependent variable.

Predictor	B	SE	Standardized β	95% CI	*p* Value
LPS	0.445	0.126	0.498	0.191–0.699	0.001

Abbreviations: LPS, lipopolysaccharide; FMD, flow-mediated dilation.

**Table 3:** Multivariable linear regression analysis with sNOX2-dp as the dependent variable.

Predictor	B	SE	Standardized β	95% CI	*p* Value
LPS	−0.148	0.034	−0.572	−0.271–−0.078	<0.001

Abbreviations: LPS, lipopolysaccharide; sNOX2-dp, soluble NOX2-derived peptide.

**Table 4:** Multivariable linear regression analysis with LPS as the dependent variable.

Predictor	B	SE	Standardized β	95% CI	*p* Value
FMD (%)	−1.347	0.455	−0.347	−2.270–−0.424	0.005
H_2_O_2_ (µM)	0.650	0.155	0.445	0.336–0.965	<0.001
Zonulin (ng/mL)	5.848	2.309	0.298	1.165–10.531	0.016

Adjusted R^2^ = 0.585. Dependent variable: LPS (pg/mL). Abbreviations: LPS, lipopolysaccharide; H_2_O_2_, hydrogen peroxide; FMD, flow-mediated dilation.

## Data Availability

The raw data supporting the conclusions of this article will be made available by the authors upon request.
